# Occupational position, work stress and depressive symptoms: a pathway analysis of longitudinal SHARE data

**DOI:** 10.1136/jech-2014-205206

**Published:** 2015-02-03

**Authors:** H Hoven, M Wahrendorf, J Siegrist

**Affiliations:** 1 Faculty of Medicine, Senior Professorship on Work Stress Research, University of Duesseldorf, Duesseldorf, Germany; 2 Centre for Health and Society, Institute for Medical Sociology, University of Duesseldorf, Duesseldorf, Germany

**Keywords:** Work stress, SOCIAL CLASS, SOCIAL INEQUALITIES, DEPRESSION

## Abstract

**Background:**

Several studies tested whether stressful work mediates the association between socioeconomic position (SEP) and health. Although providing moderate support, evidence is still inconclusive, partly due to a lack of theory-based measures of SEP and work stress, and because of methodological limitations. This contribution aims at overcoming these limitations.

**Methods:**

We conduct pathway analysis and investigate indirect effects of SEP on mental health via stressful work. Data are derived from the first two waves of the ‘Survey of Health, Ageing and Retirement in Europe’ (SHARE) with information from employed men and women aged 50–64 across 11 European countries (N=2798). SEP is measured according to two alternative measures of occupational position: occupational class (focus on employment relations) and occupational status (focus on prestige). We assess work stress according to the effort-reward imbalance and the demand-control model (wave 1), and we use newly occurring depressive symptoms as health outcome (wave 2).

**Results:**

Effort-reward imbalance and, less consistently, low control mediate the effect of occupational class and occupational status on depressive symptoms.

**Conclusions:**

Our findings point to two important aspects of work stress (effort-reward imbalance and low control) in explaining socioeconomic differences in health. Further, we illustrate the significance of two alternative dimensions of occupational position, occupational class and occupational status.

## Introduction

Research has produced solid evidence on social inequalities in health among working populations in modern societies. Overall, these inequalities in morbidity and mortality appear as social gradients across the whole of a society, leaving those in more disadvantaged socioeconomic positions (SEPs) at higher risk of poor physical and mental health.^1–4^ Similar social gradients were documented for major employment and working conditions, where people with lower SEPs are more often exposed to disadvantageous conditions, such as precarious employment including job instability,^5^ health-adverse chemical exposures^6^ and stressful psychosocial work environments.^7^ ^8^ The question of how these two gradients are intertwined, and specifically whether and to what extent health inequalities can be attributed to adverse working and employment conditions, is still debated.^9^ ^10^ This latter assumption claims that SEP exerts an indirect effect on health through adverse psychosocial working conditions,^11^ and that occupational position is the most appropriate indicator of SEP in this context.^9^

Yet, empirical support of this hypothesis is inconsistent up to now.^12^ Scarcity of prospective data, limitations of applied statistical analyses, and lack of consistency in defining and measuring, predicting and mediating constructs are among the reasons for this inconclusive state of the art.^12^ In this paper we set out to test the mediation hypothesis: (1) by analysing a large data set of a longitudinal study with the help of pathway analysis (see Methods section), (2) by introducing two alternative, well-grounded measures of occupational position (see below) and (3) by defining a stressful psychosocial work environment in terms of two established models of work stress (see below).

In this study we use two measures of occupational position. The first measure is occupational class, as measured by the internationally used Erikson-Goldthorpe-Portocarero (EGP) scheme.^13^ This scheme classifies occupations based on specific aspects under which a person performs work on the labour market, or, more specifically, based on existing ‘employment relations’. Thereby, the most basic distinction is between employers, self-employed and employees. Employees are further differentiated according to the nature of employment contract they have. The degree to which the work can be monitored and the level of required skills for performing the job are the two core aspects in that respect, leading to the distinction between ‘labour’ (easily monitored and low skill specificity) and ‘service’ contracts (difficult to monitor and high skill specificity). In sum, the EGP scheme considers important aspects of ‘employment relations’ with consequences for the individual’s income, job stability and the existing influence the worker has on the labour market.

The second measure of occupational position is occupational status, as measured by the Standard International Occupational Prestige Scale (SIOPS).^14^ In contrast to occupational class, it focuses on the general reputation or prestige assigned to an occupation, where each job is assigned a prestige value on a continuous scale. It is thus a measure indicating to what extent a person holding a particular occupation is generally esteemed or reputed in a given society. Thus, aspects of social status and its appreciation are at the core of this approach, whereas employment relations, social influence and power define the core notion of occupational class.

Concerning the mediating construct, we measure work stress in terms of two internationally-established theoretical models, the demand-control model^15^ and the effort-reward imbalance model.^16^ The former model identifies stressful work in terms of high demands in combination with low control (low decision latitude), whereas the latter model claims that an imbalance between high efforts spent and low rewards received in turn adversely affects health. Rewards include money, promotion prospects including job security, and recognition. The two models complement each other, as the first one focuses on distinct task characteristics and the role of job control, whereas the second posits that violations of reciprocal exchange between employees and employers/managers matter most, emphasising the role of reward. A large number of investigations confirmed that both models explain elevated risks of several stress-related physical and mental disorders, including depressive symptoms, the health outcome of the current study.^17–19^

In short, using two different occupational classifications and two established models of work stress, this paper investigates if work stress mediates the association between occupational position and elevated risks of depressive symptoms. As both predicting and mediating constructs are based on complementary, equally important aspects of occupational position and of work stress in modern economies, the analysis may provide a more comprehensive case as compared to previous research.

## Methods

### Data source

Data are obtained from the first two waves of the Survey of Health, Ageing and Retirement in Europe (SHARE, Release 2.5).^20^ SHARE is the first longitudinal and cross-national research project collecting comparable data on occupational position, working conditions and health among people aged 50 and older in Europe. The survey started in 2004–2005 in 11 countries (Sweden, Denmark, Germany, The Netherlands, Belgium, France, Switzerland, Austria, Italy, Spain and Greece), with on-going waves of data collection at 2 year intervals (wave 2: 2006–2007). In each country, data collection is based on probability household samples (either drawn as simple random selection or multistage random selection) where all people aged above 50 years plus their partners were interviewed using Computer Assisted Personal Interviews (CAPI). At study onset the household response rate was 61% for the total sample, with rates ranging from 81% in France to 39% in Switzerland (response rates above 50% in eight countries). This is above average compared to other European Surveys.^21^ With regard to attrition rates, 28% were lost between wave 1 and 2 (see references above for more details on SHARE). Ethical approval for SHARE was obtained by the institutional review board at University of Mannheim, Germany. In our study, all exposure variables are derived from wave 1, whereby data on the outcome variable (depressive symptoms) are taken from wave 2 according to the proposed longitudinal perspective.

### Study sample

For the analyses, we use the longitudinal sample of men and women who participated at wave 1 and wave 2 (N=18 742) and conduct additional restrictions: first, we limit the longitudinal sample to those who were employed at both waves (N=4304). This serves the objective of investigating the effects of occupational position and work stress on depressive symptoms at wave 2, and excludes those who are no longer in employment at wave 2 (22% of people who worked at wave 1). Second, because respondents aged 65 or older may have had more favourable working conditions (‘healthy worker effect’), the sample is additionally restricted to men and women aged 50–64 in wave 1. Third, to reduce the risk of reverse causality, individuals with increased depressive symptoms at wave 1 are not included either. These restrictions result in a final sample with full available data of 1658 men and 1140 women (N=2798).

### Measurement

*Occupational position:* SHARE data include a description of workers’ employment situation with detailed information on respondents’ occupation as classified by the International Classification of Occupation (4-digit ISCO-88 code).^22^ On this basis, two measures of occupational position were derived as described in the Introduction: (1) respondents’ occupational class (based on the EGP-scheme) and (2) occupational status (based on the SIOPS scale). In the case of occupational class, occupations were regrouped into four categories: (1) ‘very advantaged’ upper service class (EGP I), (2) ‘advantaged’ lower service class (EGP II), (3) ‘disadvantaged’ routine non-manuals and small proprietors (EGP III, IVab) and (4) ‘very disadvantaged’ manual supervisors, skilled and unskilled manual workers (EGP IVc, V, VI, VII). Occupational status was measured by the SIOPS prestige scale,^14^ which assigned a prestige value to each job based on the ISCO code, with higher values indicating higher status. Again, people were regrouped into four categories ranging from ‘very advantaged’ to ‘very disadvantaged’ (based on country-specific quartiles). In sum, these two categorical measures of occupational position reflect complementary, but conceptually different classifications.

### Work stress

Work stress was measured by abbreviated versions of original scales of the demand-control model and the effort-reward imbalance model. Given the constraints of a multidisciplinary approach, the inclusion of the full questionnaires was not possible in SHARE. Thus, items of the two work stress models were selected on the basis of psychometric properties. With regard to the demand-control model, the measurement was restricted to the control dimension, given the evidence that the explanatory contribution of ‘control’ exceeded the contribution of ‘demand’ in several landmark studies.^23^ ^24^ ‘Control’ was measured by the sum score of two Likert-scale items, with higher scores indicating lower control at work and a range from 2 to 8. To measure effort-reward imbalance, 2 of 6 items measuring ‘effort’ and 5 of 11 items assessing ‘reward’ at work were used. ‘Effort-reward imbalance’ was then calculated by dividing the sum score of the ‘effort’ items (nominator) by the sum score of the ‘reward’ items (adjusted for number of items; denominator). This results in a sum score ranging from 0.25 to 4, where higher values are related to higher levels of work stress. In previous analyses, both measures have successfully been associated with mental health.^25^ To enable comparisons of estimated coefficients, both scales were standardised before inclusion into multivariate analyses.

### Depressive symptoms

Our measure of mental health is a binary indicator of increased depressive symptoms, as measured by the EURO-D depression scale.^26^ The EURO-D depression scale includes 12-items for measuring number of depressive symptomatology in general population surveys. For our analyses we used a binary indicator of increased symptoms (more than 3). This was done to capture clinically relevant conditions of depressive symptoms.^27^ While this indicator may not meet the standards of a clinically-based diagnosis of depression, it was nevertheless shown to be a valid and consistent indicator of elevated levels of depressive symptoms in a cross-European study.^26^

### Additional variables

Age, gender and country affiliation were additionally included, mainly as confounders within pathway analyses. An overview of all measures is presented in table 1.

**Table 1:** Sample characteristics, N=2798

Variable	Categories or range	Complete case % or mean(SD)	N
Sex	Male	59.26	1658
	Female	40.74	1140
Age	50–64	54.76 (3.30)	
Depressive symptoms	No	91.57	2562
	Yes	8.43	236
ERI	0.25–4.0	0.98 (0.40)	
Low control	2–8	4.00 (1.38)	
Occupational class	Very advantaged	24.80	694
	Advantaged	25.16	704
	Disadvantaged	24.62	689
	Very disadvantaged	25.41	711
Occupational status	Very advantaged	20.05	561
	Advantaged	27.56	771
	Disadvantaged	25.48	713
	Very disadvantaged	26.91	753

All measures except depressive symptoms (wave 2) are taken from wave 1.

ERI, effort-reward imbalance.

### Statistical analysis

Following a general description of the sample (table 1), average levels of work stress and percentages of newly occurring depressive symptoms for each category of the two measures of occupational position are investigated (table 2). Thereafter, pathway analyses are conducted to test the mediating hypothesis. With few notable exceptions,^28^ ^29^ previous studies applied nested regressions that compare successive models without and with mediator variables. Compared to nested regression analysis, pathway analysis confers several advantages (see refs. 30 and 31 for a detailed discussion). First, nested regression analysis is not based on a quantification of the intervening effect, but infers this effect from an attenuation of effect sizes in subsequent model tests. Pathway analysis instead focuses on the effect of interest, that is, the indirect effect via the mediator variable. Second, by using pathway analysis we can evaluate indirect effects via the mediator, even if the outcome of interest is binary (as in our study), a case that was shown to be problematic in nested regression analysis.^32^ Third, while conventional approaches require a significant association between predictor and outcome, pathway analysis can also detect indirect effects in the absence of such an association, thus minimising the possibility of false-negative findings, as proven in a number of simulation studies.^33^

**Table 2:** Average level of work stress and percentage with increased depressive symptoms by core variables: mean scores and SD, or per cent (N=2798)

Variable	Categories or range	Mean ERI (SD)	Mean low control (SD)	Percent of depressive symptoms
Sex	Male	1.00 (0.41)	4.01 (1.38)	6.39
	Female	0.95 (0.39)	4.00 (1.38)	11.40
Depressive symptoms	No	0.97 (0.40)	3.98 (1.37)	
	Yes	1.05 (0.44)	4.15 (1.46)	
Occupational class	Very advantaged	0.88 (0.34)	3.57 (1.22)	6.05
	Advantaged	0.92 (0.36)	3.76 (1.23)	10.65
	Disadvantaged	0.97 (0.38)	4.05 (1.36)	7.69
	Very disadvantaged	1.13 (0.46)	4.60 (1.47)	9.28
Occupational status	Very advantaged	0.87 (0.34)	3.58 (1.25)	7.49
	Advantaged	0.90 (0.33)	3.71 (1.19)	8.17
	Disadvantaged	1.03 (0.42)	4.11 (1.42)	8.42
	Very disadvantaged	1.08 (0.45)	4.50 (1.44)	9.43

ERI, effort-reward imbalance.

**Figure 1:**
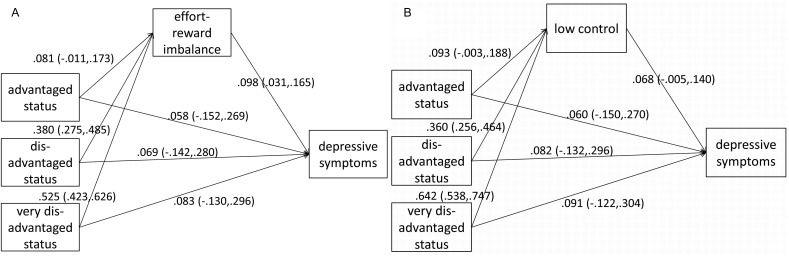
Pathway analyses of the association between occupational status, work stress (effort-reward imbalance (ERI) and low control) and increased depressive symptoms: Adjusted for country-affiliation, sex and age, N=2798.

**Figure 2:**
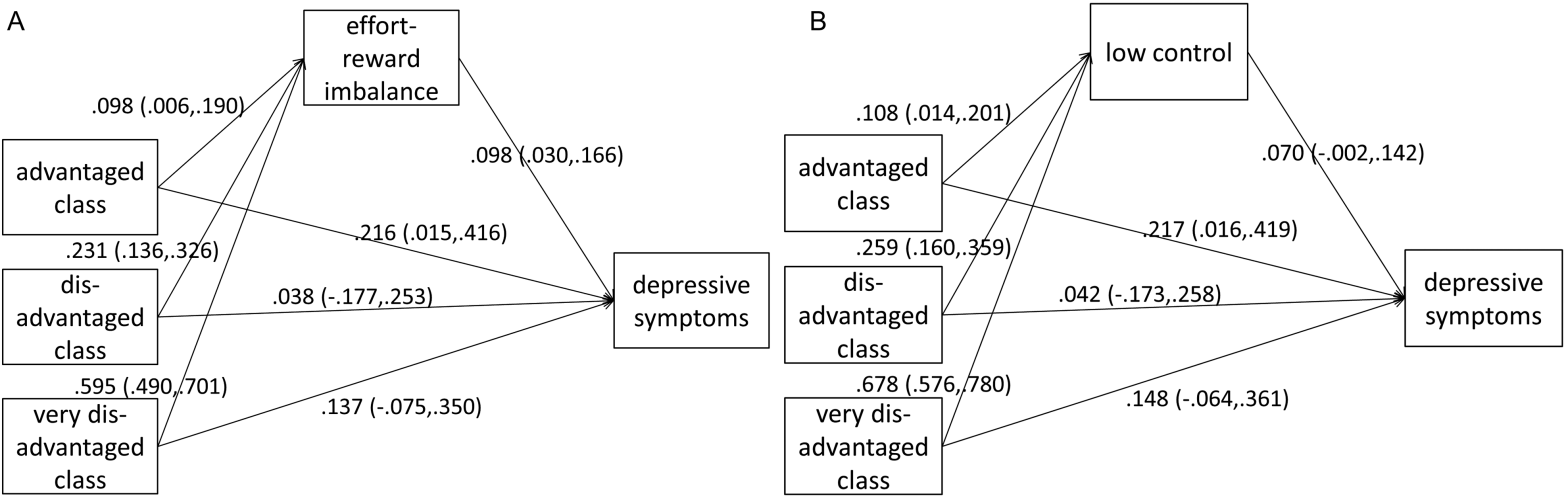
Pathway analyses of the association between occupational class, work stress (effort-reward imbalance (ERI) and low control) and increased depressive symptoms: Adjusted for country-affiliation, sex and age, N=2798.

In the Results section, findings of four pathway models are presented, two for each measure of occupational position (class and status) which, investigate the mediation effects on depressive symptoms either via low control or via effort-reward imbalance. All models were estimated with MPLUS^34^ because it allows estimating pathway models with categorical as well as continuous variables (see 34 for more details). Findings for occupational status are shown in figure 1, and findings for occupational class are displayed in figure 2. In these figures, unstandardised coefficients for each direct path within the models are presented (all adjusted for sex, age (linear) and country affiliation). These are either based on linear regressions (when studying the path between occupation and work stress), or on probit regression models (when studying effects on depressive symptoms). Finally, table 3 presents the estimation of the indirect effects. In each case, CIs are shown that are based on bias-corrected bootstrapping procedures with 5000 iterations.^35^ In addition, to summarise the key findings in a coherent and clear way, estimated indirect effects are visualised in online supplementary figure S1.

**Table 3:** Indirect effects of occupational position via work stress on depressive symptoms: unstandardised coefficients and CIs (95%)

	Indirect effects via	
	Low control	Effort-reward imbalance
Class		
Very advantaged	Ref.	Ref.
Advantaged	0.008 (0.000 to 0.023)	0.010 (0.001 to 0.024)
Disadvantaged	0.018 (0.001 to 0.042)	0.023 (0.008 to 0.045)
Very disadvantaged	0.048 (−0.002 to 0.097)	0.058 (0.018 to 0.100)
Status		
Very advantaged	Ref.	Ref.
Advantaged	0.006 (0.000 to 0.021)	0.008 (0.000 to 0.023)
Disadvantaged	0.024 (−0.001 to 0.054)	0.037 (0.012 to 0.069)
Very disadvantaged	0.043 (−0.003 to 0.090)	0.051 (0.017 to 0.090)

N=2798.

Estimates are based on pathway models, 95% CIs of indirect effects are based on bootstrapping procedure. Adjusted for sex, age and country affiliation.

ERI, effort-reward imbalance.

## Results

Sample characteristics are presented in table 1. The mean age is 55 years and the sample consists of more men than women. On average, about 8% developed increased depressive symptoms between wave 1 and wave 2. Turning to table 2, a clear social gradient of work stress is obvious, where respondents having disadvantaged occupations (either in terms of status or of class) have higher levels of work stress (either low control or effort-reward imbalance). A similar gradient is observed in case of depressive symptoms, with the exception of respondents from the lower service class (‘advantaged’) who exhibit a high level of depressive symptoms.

Figures 1 and 2 present results of pathway analysis in terms of regression coefficients for each path in respective models. These paths inform about estimated net effects after adjusting for all remaining variables. Figure 1 displays the results of pathway analyses with occupational status as a measure of occupational position. In figure 2, pathway analyses with occupational class are presented. We briefly describe figure 1A as a guide for interpretation of the remaining figures. In the paths leading from occupational status to depressive symptoms, we observe that the pattern of regression coefficients between occupational status and depressive symptoms fits with the notion of a social gradient (see table 2), although effects are non-significant when all remaining variables are considered. In addition, more disadvantaged occupational status is associated with increased levels of work stress. Furthermore, paths leading from work stress to depressive symptoms show a positive effect (slightly more pronounced in case of effort-reward imbalance (figure 1A) than in case of low control (figure 1B)). This means that higher work stress is associated with a higher probability of exhibiting depressive symptoms.

Turning to figure 2, where findings for occupational class are displayed, results are well comparable to the former ones. In line with data in table 2, a gradual association between occupational class and work stress is observed. Moreover, work stress is again linked to depressive symptoms, and—with the exception of the lower service class—a gradient between occupational class and depressive symptoms is apparent.

In a next step, indirect effects are calculated to answer the main research question. Results are given in table 3, where coefficient and CIs are indicated for both measures of occupational position, either mediated via low control or via effort-reward imbalance. Two observations deserve attention. First, the indirect effects of occupational position via work stress on depressive symptoms are generally more pronounced in the more disadvantaged occupations. This observation is of interest as it points to a potentially higher susceptibility of lower occupational positions to the adverse effects of stressful work. Second, while findings were similar for both measures of occupation, indirect effects were more consistent in the case of effort-reward imbalance as compared to low control, where effects were just below significant level in three of six cases (see online supplementary figure S1 for a visual summary of indirect effects).

## Discussion

The findings of our analyses can be summarised as follows. First, results indicate a gradual association between occupational position and levels of work stress. Moreover, both variables exert independent effects on subsequent depressive symptoms. Second, importantly, we observed an indirect effect of occupational position through work stress on depressive symptoms. This effect is consistent for the occupational class and occupational status, but is more pronounced in the case of effort-reward imbalance.

This study adds two new elements to current research testing the mediation hypothesis.^12^ First, because we used two complementary and conceptually well-grounded measures of SEP in terms of occupation, we can give a more comprehensive interpretation as compared to previous studies. In the case of occupational class, it seems likely that the observed indirect effects among lower classes (mainly employees working in low skilled jobs) are due to lower social influence, lower income and precarious employment, all components that are directly related to control at work^36^ or the experience of reward.^8^ Furthermore, results in the case of occupational status point to the importance of reputation and prestige of a job in determining its level of work stress, most likely because it increases the level of non-material reward (as an important component of the effort-reward imbalance model) and of individual autonomy (as an important component of control).^37^ Second, while most previous studies analysed the mediation hypothesis using the demand-control model and applied nested regression, this study also includes effort-reward imbalance and is based on pathway modelling. Hence, this is probably the first longitudinal study to compare two theory-based indicators of occupational position in combination with two models of work stress applying path analysis. Findings indicate that recurrent experiences of efforts at work that outmatch rewards link low occupational position with poor mental health.

Although this study profits from several strengths (longitudinal study design, large sample size, comprehensive measures of occupational position and work stress, and pathway modelling), we have to consider several limitations. First, the sample is restricted to employed men and women at older ages and, thus, any generalisation of findings do require additional studies based on younger cohorts. More specifically, there is a risk that the observed effects of work stress are biased, for example, because levels of work stress are generally higher in younger age groups where the burden of disease attributable to stressful work may be even higher.^38^ Second, while it has been hypothesised that mediation effects may differ by sex,^28^ separate analyses of men and women were not feasible due to the small number of respective subsamples. Third, the assessment of the two work stress models was incomplete and therefore we may run the risk of underestimating the effects under study.^39^ This may specifically be the case for the assessment of the demand-control model, as control at work was measured by two items only, and the demand component was not included in this data set. Thus, the slightly less consistent indirect effect in case of low control may be due to the limited assessment. In addition, the component ‘overcommitment’, reflecting the working person's intrinsic effort in the complementary work stress model, was not measured. Fourth, the health outcome of this study, newly occurring depressive symptoms, was measured by a self-report questionnaire instead of by a clinical interview conducted by experts. Yet, the previous validation studies indicate that the EURO-D scale used in this study^26^ provides fairly valid estimates of clinically relevant depressive symptoms, if compared with clinical judgments.^27^

In conclusion, the results of this study offer limited evidence that stressful work mediates the association of occupational position with newly occurring depressive symptoms among older employed men and women in different European countries. If supported by further research, our findings lend support to policies that aim at reducing health inequalities by improving the quality of work among less privileged occupational groups.

**What is already known on this subject**

- Several cohort studies tested a mediating role of stressful work, most often measured by high demand combined with low control, in associations between different indicators of socioeconomic position and health.
- Results provide moderate support, but are confined to the absence of theory-based measures of predicting and mediating variables, and the use of nested regressions.

**What this study adds**

- By applying pathway modelling, direct and indirect effects of occupational position and stressful work on depressive symptoms, the health outcome, are estimated in a large sample of men and women.
- We assess occupational position by two theory-based indicators, class and status, and we measure work stress by low control and effort-reward imbalance.
- Results show that low control and effort-reward imbalance both mediate effects of occupational position on depressive symptoms, and we illustrate the importance of two core constructs of social stratification, occupational status and occupational class.

## Supplementary Material

Web supplement
