# Supplementary material for: Occupational position, work stress and depressive symptoms: a pathway analysis of longitudinal SHARE data
Source: J Epidemiol Community Health. 2015 Feb 3;69(5):447–52. doi: 10.1136/jech-2014-205206 (PMC4413688; doi:10.1136/jech-2014-205206)

Supplementary Figure 1: Indirect effects of occupational position via work stress on depressive symptoms: Unstandardised coefficients and confidence intervals (95%). N=2798.

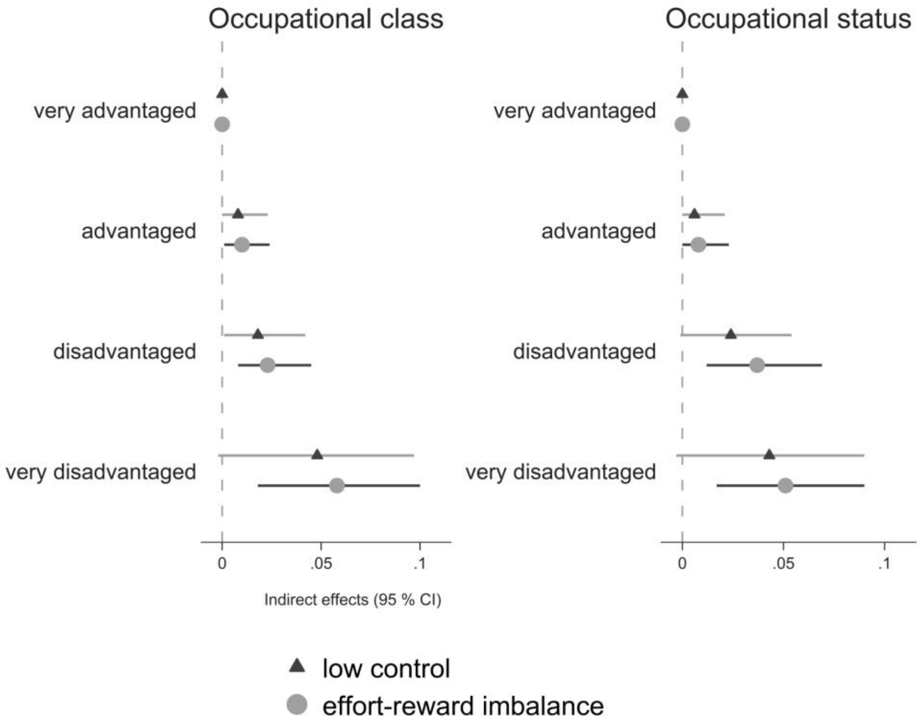

Supplement: Web supplement [file jech-2014-205206-s1.pdf]
